# Chemical Composition and Antioxidant Properties of Common and Lemon Verbena

**DOI:** 10.3390/antiox11112247

**Published:** 2022-11-15

**Authors:** Milena Polumackanycz, Spyridon Alexandros Petropoulos, Mikel Añibarro-Ortega, José Pinela, Lillian Barros, Alina Plenis, Agnieszka Viapiana

**Affiliations:** 1 Department of Analytical Chemistry, Medical University of Gdansk, Gen. J. Hallera 107, 80-416 Gdansk, Poland; 2 Department of Agriculture, Crop Production and Rural Environment, University of Thessaly, Fytokou Street, 38446 Volos, Greece; 3 Centro de Investigação de Montanha (CIMO), Instituto Politécnico de Bragança, Campus de Santa Apolónia, 5300-253 Bragança, Portugal; 4 Laboratório Associado para a Sustentabilidade e Tecnologia em Regiões de Montanha (SusTEC), Instituto Politécnico de Bragança, Campus de Santa Apolónia, 5300-253 Bragança, Portugal

**Keywords:** nutritional composition, phenolic compounds, antioxidant activity, bioactive properties, tocopherols, organic acids, *Verbena officinalis* L., *Aloysia citrodora* L.

## Abstract

The nutritional profiles of common and lemon verbena leaves were analyzed (proximate constituents, free sugars, organic acids, tocopherols, and fatty acids) and the leaves were prepared in hydromethanolic and aqueous (decoctions and infusions) extracts. The phenolic compound composition and antioxidant activity (2,2-Diphenyl-1-picrylhydrazyl (DPPH); 2,2′-azino-bis(3-ethylbenzothiazoline-6-sulfonic acid (ABTS), ferric-reducing antioxidant power (FRAP); and cupric-reducing antioxidant capacity (CUPRAC) assays) of the extracts were characterized. The nutritional composition varied between the studied species, with lemon verbena showing higher amounts of protein, ash, and fat than common verbena, whereas the opposite trend was recorded for the dietary fiber content. The main free sugars detected in both species were fructose, glucose, and sucrose, which were present in higher amounts in the common verbena samples. Succinic acid was the most abundant organic acid in both species while high amounts of oxalic acid were detected in lemon verbena. The main fatty acids in both species were α-linolenic, palmitic, and linoleic acid. Regarding the phenolic compound content, the extracts of lemon verbena presented higher amounts of total phenolic compounds (TPCs), total flavonoids (TFs) and total phenolic acids (TPAs) than the common verbena extracts while the aqueous extracts (infusions and decoctions) were richer in TPCs, TFs, and TPAs than the hydromethanolic ones in both species. Nine phenolic compounds were identified and quantified, including seven phenolic acids and two flavonoids. The lemon verbena samples were characterized by higher antioxidant activity compared to the common verbena samples while the aqueous extracts showed higher antioxidant efficacy than the hydromethanolic ones. In conclusion, both species showed promising results in terms of the nutritional value, chemical composition, and antioxidant activities, which were positively correlated with the phenolic compound contents. Moreover, the extraction protocol may affect the chemical composition and bioactive properties of both species, with aqueous extracts showing better results than hydromethanolic ones.

## 1. Introduction

Since ancient times, medicinal and aromatic plants have been used in traditional and folk medicine to heal different human ailments [1]. In recent years, an increasing interest in the biological properties of medicinal plants has been observed, which has focused on the discovery and evaluation of therapeutic potential and the identification of the major bioactive compounds and possible synergisms [2].

The Verbenaceae family comprises over 100 genera and about 2000 species with a wide geographical distribution, including the tropical, subtropical, and temperate regions of the world [3]. This family includes aromatic species that have been used since ancient times in ethnobotany, such as beverages, food, seasoning, and remedies in traditional medicine [4,5].

Phenolic compounds are one of the most studied classes of bioactive molecules by the scientific community due to their antioxidant activity and beneficial health effects [6]. For this reason, they are considered the most bioactive ingredients of medicinal plant infusions and decoctions [7,8]. When consumed on a daily basis, they may promote human well-being since several studies have confirmed an important inhibitory activity against several serious chronic diseases, such as cancer, Alzheimer’s disease, and diabetes, among others [9]. However, their composition varies considerably and can be affected by several pre-harvest conditions, mainly the maturation state [8,10,11].

The antioxidant activity of phenolic compounds exhibited by plant extracts and essential oils has been crucial to the application of these compounds in the food industry as natural preservatives, thus increasing the shelf-life of several foodstuffs [12,13]. Phenolic compounds have also been used as ingredients in processed foods to enhance their functional properties and provide consumer health benefits through their radical scavenging activities [14]. For example, *Rosmarinus officinalis* L. extract increases the antioxidant activity in cottage cheese while herbal mixtures containing *Laurus nobilis* L., *Curcuma longa* L., and *Zingiber officinale* Roscoe showed improved nutritional value and total phenolic and flavonoid contents, and a reduced bacterial content in extruded corn snacks [15].

*Verbena officinalis* L., commonly known as vervain or common verbena, has a wide geographical distribution from South America to North America, northern Africa, Europe, Asia, and Australia [16]. This herb has been used for many years as a health supplement in herbal medicine [17,18]. The latest studies on the species have shown a wide variety of pharmacological effects, including antioxidant, antibacterial, antiphlogistic, antifungal, anti-inflammatory, antiproliferative, analgesic, insecticidal, and gastroprotective properties [19,20]. It is traditionally used to treat nervous system disorders, such as depression, insomnia, and anxiety [21], while research data indicate that *V. officinalis* can be applied in the treatment of liver diseases, hepatitis and cholecystitis, in menstrual disorders, and in breast-feeding mothers to stimulate lactation [21]. The species is also known for its diuretic effect, being used in kidney and urinary bladder diseases, while it is also administered to treat fever accompanying colds and as a supportive agent in malaria and rheumatism [21,22]. The main constituents of the aerial parts of *V. officinalis* include verbenalin, hastatoside, and aucubin while the dominant phenylpropanoid glycosides are verbascoside, isoverbascoside, and eukovoside [23,24]. Moreover, several phenolic acids have been identified, including chlorogenic, ferulic, protocatechuic, and rosmarinic acids and dicaffeoylquinic acid derivatives [25]. The main flavonoids are kaempferol, apigenin, luteolin, scutellarein, and pedalitin while essential oil is dominated by terpenoids [19,26,27,28]. Other compounds that have been detected are sterols and carbohydrates [29].

*Aloysia citrodora* Paláu (syn. *Aloysia triphylla* (L’Hér.) Britton), commonly known as lemon verbena, is an annual plant that originates in South America and is cultivated throughout the Mediterranean and North Africa [30]. It has traditionally been consumed as a herbal tea for the treatment of cold and fever, influenza, colic, diarrhea, spasms asthma, anxiety, insomnia, and indigestion cases [31,32]. Moreover, this plant is popularly used as a tincture or essential oil for dermal disorders [33]. Studies on *A. citrodora* have shown its potent biological effects, such as antioxidant, antifungal, anti-inflammatory, and antimicrobial properties [34,35]. Its leaves are highly appreciated for their characteristic lemony scent and are commonly used in many food preparations, such as fish and poultry dishes, vegetable marinades, salad dressings, jams, puddings, beverages, and sorbets [36]. The plant contains large amounts of phenolic compounds such as phenylpropanoids (mainly verbascoside), flavonoids, lignans, and tannins, and a variety of other non-phenolic compounds [5,37,38,39,40]. Moreover, the essential oil of *A. citrodora* leaves is dominated by the citral isomers, geranial and neral [41,42], while several studies have demonstrated that the oil composition is affected by numerous factors such as the genotype, environmental factors, and growth conditions [43].

Several studies refer to the chemical composition of common and lemon verbena; however, there are scarce literature reports about the constituents of aqueous preparations although they are attributed with important biological activities such as antigenotoxic effects and protection against genetic damage of lemon verbena infusion [44] or the neuroprotective [29,45] and hypnotic effects of the aqueous extract of verbena [46]. Moreover, some studies have reported the antioxidant properties of the species; however, they mostly refer to essential oils [34,47,48]. Nevertheless, as far as we know, there are no reports available on verbena aqueous extracts obtained by decoctions.

To the best of the authors’ knowledge, there is scarce information on the chemical composition and antioxidant activity of common and lemon verbena aqueous extracts. Considering the wide use of different preparations of infusions of medicinal plants by the population, the aim of this study was to characterize and compare the phenolic compound composition of common and lemon verbena aqueous (infusions and decoctions) and hydromethanolic extracts and reveal the relationship between the bioactive compound contents and the antioxidant activity determined by four methods (DPPH, ABTS, FRAP, and CUPRAC). Given the food applications of these plant species, their nutritional composition (proximate constituents, free sugars, organic acids, fatty acids, and tocopherols) was also investigated.

## 2. Materials and Methods

### 2.1. Samples

Common and lemon verbena samples were purchased from a local Polish company (NATUR-VIT, Poland), a certified collector and producer of medicinal herbs, in the form of dried herbs in February 2021. The samples of common and lemon verbena were collected by NATUR-VIT in Kopernia, Pińczow (50°31′13″ N 20°31′35″ E) from 70 plants growing in 2 different grasslands of about 3 ha for each plant. The gathered material of each verbena was mixed and made into a unique sample. Moreover, the samples were clean products with monitored parameters of pesticides, herbicides, heavy metals, and radioactivity. Each sample was pulverized in a water-cooled Knifetec1095 grinder (Foss Tecator, Höganäs, Sweden) to a fine dried powder (20 mesh) and the homogenized samples were stored in a light-proof desiccator.

### 2.2. Standards and Reagents

Nine phenolic standards including gallic acid (GA), syringic acid (SRA), *p*-coumaric acid (*p*CA), ferulic acid (FA), sinapic acid (SNA), cinnamic acid (CNA), protocatechuic acid (PRA), rutin (RUT) and quercetin (Q), 2,2-diphenyl-1-picrylhydrazyl (DPPH reagent), 2,2-azinobis (3-ethylbenzothiazoline-6-sulfonic acid) diammonium salt (ABTS reagent), 4-chloro-7-nitrobenzofurazan (NBD-Cl), ammonium acetate, and neocuproine were purchased from Sigma-Aldrich (St. Louis, MO, USA). In all cases, the purity of the standards exceeded 98%. Aluminum chloride (AlCl_3_) was obtained from AcrossOrganics (Morris Plains, NJ, USA) and high-performance liquid chromatography (HPLC)-grade acetonitrile (ACN) was obtained from Avantor (Central Valley, PA, USA) while the other reagents were obtained from POCh (Gliwice, Poland). The redistilled water was prepared by triple distillation of water in a Destmat^®^ Bi-18 system (Heraeus Quarzglas, Hanau, Germany).

### 2.3. Nutritional Value

#### 2.3.1. Proximate Composition and Energy

The protein, fat, ash, and dietary fiber contents were determined following the Association of Official Analytical Chemists (AOAC) methods [49]. Briefly, the crude protein content (N × 6.25) was estimated by the macro-Kjeldahl method (AOAC 920.152) using an automatic distillation and titration unit (Pro-Nitro-A, JP Selecta, Barcelona); the crude fat content was determined by Soxhlet extraction with petroleum ether (AOAC 920.85); the ash content was determined by incineration in a muffle furnace at 550 ± 15 °C (AOAC 940.26); and the total dietary fiber content was determined by an enzymatic-gravimetric method (AOAC 985.29). Carbohydrates were estimated by difference. The results were presented as g per 100 g of plant material on a dry weight (dw) basis.

The energy value was calculated using the following conversion factors: 9 kcal/g for fat, 4 kcal/g for proteins and carbohydrates, and 2 kcal/g for fiber and the results were expressed in kcal per 100 g of plant material.

#### 2.3.2. Composition in Free Sugars and Organic Acids

Free sugars were analyzed in an HPLC system (Knauer, Smartline system 1000, Berlin, Germany) coupled to a refraction index (RI) detector as previously described [50]. Briefly, the samples (1 g) were spiked with melezitose (internal standard, 5 mg/mL) and extracted with 80% ethanol at 80 °C for 90 min. After filtration, the supernatants were concentrated under reduced pressure, defatted with ethyl ether, and concentrated again. The residues were then dissolved in 5 mL of water and filtered through 0.2 µm nylon filters for HPLC analysis. Chromatographic separation was achieved with a Eurospher 100-5 NH_2_ column (4.6 mm × 250 mm, 5 mm, Knauer) using acetonitrile/water 70:30 (*v/v*) as the mobile phase at a 1 mL/min flow rate. Free sugars were identified by chromatographic comparisons with authentic standards and were quantified based on the internal standard concentration. The results were expressed in g per 100 g of plant material.

Organic acids were analyzed in an ultra-fast liquid chromatography (UFLC) system (Shimadzu 20A series, Kyoto, Japan) coupled to a photodiode array detector (PDA) as previously described [51]. Briefly, each sample (1 g) was stirred with 25 mL of meta-phosphoric acid for 45 min and filtered, first through Whatman No. 4 filter paper and then through 0.2 µm nylon filters. Chromatographic separation was achieved in reverse phase on a C18 column (250 mm × 4.6 mm, 5 µm; Phenomenex, Torrance, CA, USA) using 3.6 mM sulfuric acid as the mobile phase at a 0.8 mL/min flow rate. Detection was performed on a photo diode array at 215 and 245 nm. The detected compounds were identified and quantified by chromatographic comparison of the peak area with calibration curves obtained from commercial standards. The results were expressed in g per 100 g of plant material.

#### 2.3.3. Composition in Fatty Acids and Tocopherols

The crude fat obtained by Soxhlet was mixed with 5 mL of methanol: sulfuric acid 95%: toluene 2:1:1 (*v*/*v*/*v*) and transesterified by stirring (160 rpm) in a water bath at 50 °C for 12 h. The fatty acid methyl ester (FAME) mixture recovered by phase separation (using deionized water and diethyl ether) was filtered through a 0.2 µm nylon filter and analyzed in a YOUNG IN Chromass 6500 Gas Chromatography System (YL Instruments, Anyang, Korea) equipped with a flame ionization detector (FID) as previously described [50]. Separation was carried out on a Zebron™ ZB-FAME column (30 m × 0.25 mm, 0.20 µm) using an oven temperature program as follows: 100 °C initial temperature, held for 2 min, increase by 10 °C/min to 140 °C, followed by a 3 °C/min ramp to 190 °C, a 30 °C/min ramp to 260 °C, and held for 2 min. Hydrogen was used as the carrier gas at a 1.1 mL/min flow rate. The detected fatty acids were identified by chromatographic comparison of the retention times of the sample FAME peaks with those of the standard 47885-U. The results were expressed as the relative percentage of each fatty acid.

Tocopherols were analyzed in the HPLC system coupled to a fluorescence detector (FP-2020, Jasco, Easton, MD, USA) programmed for excitation at 290 nm and emission at 330 nm, following a previously described analytical procedure [52]. The samples (500 mg) were spiked with tocol (internal standard, 50 μg/mL) and extracted with methanol, hexane, and a saturated NaCl solution. After the upper layer was collected, the extraction was repeated twice with hexane. The extracts were dried under a nitrogen stream, redissolved in *n*-hexane, and filtered through 0.22 μm syringe filters. Chromatographic separation was carried out in normal phase on a Polyamide II column (250 × 4.6 mm, 5 μm; YMC, Kyoto, Japan) using *n*-hexane/ethyl acetate (70:30, *v/v*) as the mobile phase at a 1 mL/min flow rate. The detected compounds were identified by chromatographic comparisons with authentic standards and quantified using the internal standard method. The results were given as mg per 100 g of plant material.

### 2.4. Non-Nutritional Composition

#### 2.4.1. Extract Preparation

For the hydromethanolic extracts, 1 g of each sample was sonicated with 4 mL of methanol-water mixture (80:20, *v*/*v*) for 15 min at 25 °C using an ultrasonic bath (Emag, Salach, Germany). The suspension was centrifuged in an EBA-20S centrifuge (Hettich, Tuttlingen, Germany) for 5 min at 8000 rpm and the supernatant was transferred into a volumetric flask. This procedure was repeated twice, and the obtained extracts were combined and diluted up to 20 mL with a mixture of methanol-water (80:20; *v*/*v*).

For the preparation of the infusions, each sample (1 g) was added to 100 mL of boiling distilled water and left to stand at room temperature for 15 min, and then filtered through the Whatman filter paper no. 113 (Sigma-Aldrich, St. Louis, MO, USA).

For the preparation of the decoction, each sample (1.0 g) was added to 100 mL of boiling distilled water, heated on a heating plate, and boiled for 10 min. The mixture was left to stand for 10 min and then filtered through a Whatman filter paper no. 113 (Sigma-Aldrich, St. Louis, MO, USA).

Before HPLC analysis, hydromethanolic and water extracts (infusions and decoctions) of common and lemon verbena were filtered through a 0.25 μm nylon filter film (Mecherey, Nagel, Germany) and 20 μL of the filtrate was injected into the HPLC system.

#### 2.4.2. Analysis of Phenolic Compounds

Phenolic compounds were determined using an HPLC LaChrom system (Merck, Darmstadt, Germany) with a Hypersil Gold C18 column (250 × 4.6 mm; 5 μm) (Thermo Scientific, Runcorn, UK). The column was thermostatically controlled at 45 °C. The system consisted of an L-7420 UV-Vis detector, L-7200 autosampler, and L-7360 thermostat. The flow rate was set at 0.8 mL/min, the injection volume was 20 μL, and the total run time was 50 min. Solvent A (0.5% acetic acid in acetonitrile) and solvent B (0.5% acetic acid in water) were used as a mobile phase. A gradient program was chosen as follows: 0–10 min, linear 5–10% A; 10–35 min, linear 10–43% A; 35–40 min, linear 43–63% A; 40–45 min, isocratic 65% A; 45–50 min, linear 63–5% A. The detection wavelengths were set at 280 (GA, SRA, PRA, SNA, and CNA), 320 (*p*CA and FA), and 370 nm (RUT and Q). The identification of the phenolic compounds was based on the comparison of the retention time and UV-Vis spectrum of their standards. Additionally, a selected sample was spiked with the standard compounds and analyzed again.

The HPLC method developed for the quantitation of the phenolic compounds was validated by the linear range, limit of detection (LOD), limit of quantitation (LOQ), precision, and accuracy according to a previously described procedure [53]. The validation parameters for the HPLC procedure are listed in Table 1. Detailed inspection of the data shows that the precision of the HPLC procedure was acceptable; the coefficient of variation (CV) values ranged between 0.32 and 3.76% and 0.42 and 8.12% for the intra- and inter-day variations, respectively. For the stability test, the retention CV was lower than 1.5% for the peak area and 0.6% for the retention time. Apart from this, the peak areas and retention times of the phenolic compounds were found to be sufficiently stable over 48 h.

#### 2.4.3. Total Phenolic Compound (TPC), Total Flavonoid (TF), and Total Phenolic Acid (TPA) Content

The total phenolic compound (TPC) content was estimated by the Folin–Ciocalteu method [54] with some modification. Briefly, 0.2 mL of the Folin–Ciocalteu reagent was added to 0.1 mL of common and lemon verbena extracts and, after 2 min, 7% Na_2_CO_3_ (*w*/*v*) (2 mL) was added. After shaking, the solution was kept at rest for 1 h at room temperature. The absorbance was determined by spectrophotometry at 760 nm after the time of incubation. TPC was calculated as gallic acid using the following equation based on the calibration curve: y = 11.30x + 0.016, r^2^ = 0.989, where x is the absorbance and y is the gallic acid concentration. The obtained results expressed as mg of gallic acid equivalents (GAE) per 1 g dry weight (DW) of each extract.

The total flavonoid (TF) content was estimated according to the *European Pharmacopoeia* [55]. Briefly, 1 mL of common verbena and 0.5 mL of lemon verbena extract were mixed with 0.1 mL of 5% AlCl_3_ (*w*/*v*) solution and with 1.4 mL of acetic acid and methanol (1:19) mixture. The absorbance was measured at 425 nm after 30 min of incubation in the dark. The TF content was extrapolated using the linear equation: y = 28.14x − 0.019, r^2^ = 0.992, where x is the absorbance and y is the concentration of quercetin. The results were expressed as μg of quercetin equivalents (QE) per 1 g DW of extract.

The total phenolic acid (TPA) content was assessed using Arnov’s reagent as described in the *Polish Pharmacopoeia VI* [56]. Briefly, 1 mL of diluted common verbena and 0.5 mL of diluted lemon verbena extract were mixed with 0.2 mL of hydrochloric acid (0.5 M), 0.2 mL of Arnov’s reagent, and 0.2 mL of sodium hydroxide (1 M). The absorbance was measured at 490 nm and the TPA content was calculated on the basis of the calibration curve: y = 18.02x − 0.068, r^2^ = 0.995, where x is the absorbance and y is the caffeic acid concentration. The results were expressed as mg of caffeic acid equivalents (CAE) per 1 g DW of extract.

### 2.5. Antioxidant Activity

#### 2.5.1. DPPH Radical Scavenging Activity

The DPPH assay was performed in accordance with a modified method of Tuberoso et al. [57]. Briefly, 0.05 mL of diluted extract was added to 2.8 mL of DPPH solution (100 μmol/L) and after 10 min, spectrophotometric measurement was carried out at 517 nm. A calibration curve was prepared with Trolox, and the equation of the curve was y = −6.90x + 0.78, r^2^ = 0.988, where x is the absorbance and y is the concentration of Trolox. The results were expressed in mg of Trolox. The equation of the curve was y = −6.90x + 0.78, r^2^ = 0.988, where x is the absorbance and y is the concentration of Trolox. equivalents (TEs) per 1 g DW of extract.

#### 2.5.2. ABTS Radical Scavenging Activity

For the ABTS assay, the procedure followed the method of Arnao et al. [58] with some modifications. A volume of 0.025 mL of each diluted extract was added to 2 mL of ABTS solution and after 6 min, spectrophotometric measurement was carried out at 734 nm. A calibration curve was prepared with Trolox, and the equation of the curve was y = −5.14x + 0.72, r^2^ = 0.985, where x is the absorbance and y is the concentration of Trolox. The results were expressed in mg of Trolox equivalents (TE) per 1 g DW of extract.

#### 2.5.3. FRAP Assay

The FRAP assay was performed using the method proposed by Benzie and Strain [59]. A volume of 0.025 mL of each diluted extract was added to 2.25 mL of FRAP solution and, after 30 min, spectrophotometric measurement was carried out at 593 nm. A calibration curve was prepared with ferrous sulphate and the equation of the curve was y = 0.59x − 0.05, r^2^ = 0.992, where x is the absorbance and y is the concentration of ferrous sulphate. The results were expressed in mmol of ferrous ion equivalents per 1 g DW of extract (mmol Fe^2+^/g DW).

#### 2.5.4. CUPRAC Assay

The CUPRAC assay was performed according to the method of Apak et al. [58] with some modifications. A volume of 0.05 mL of diluted extract was added to 1 mL of neocuprine ethanolic solution (7.5 mM), 1 mL of copper chloride solution (0.01 M), and 1 mL of ammonium acetate buffer solution (pH = 7.00). After 30 min, spectrophotometric measurement was carried out at 495 nm. A calibration curve was prepared with ascorbic acid and the equation of the curve was y = 1.58x + 0.21, r^2^ = 0.982, where x is the absorbance and y is the ascorbic acid concentration. The results were expressed in mg ascorbic acid equivalents (AAE) per 1 g DW of extract.

### 2.6. Statistical Analysis

For common and lemon verbena, three samples were used, and all assays were carried out in triplicate. The results are expressed as mean values and standard deviations (SDs). The relationship between the phenolic composition and antioxidant activity was analyzed by Pearson correlation analysis. For the nutritional composition and fatty acid analysis, the results were analyzed by a one-way ANOVA to study the differences between the types of plant. For the remaining analyses, a two-way ANOVA was applied to study the effect of the type of the plant and the extraction as the main factors. As a significant interaction between the tested factors was observed, a one-way analysis was carried out to study differences between all the samples. Statistical analysis was performed using Statistica 10 software (StatSoft Inc., Tulsa, OK, USA).

## 3. Results and Discussion

### 3.1. Nutritional Composition

The results of the nutritional composition of the lemon and common verbena samples are presented in Table 2. Significant differences were recorded in the nutritional profile of the studied species. In particular, lemon verbena (*Aloysia citrodora*) had a higher content of proteins, ash, and fat (13.7, 9.9, and 1.90 g/100 g dw, respectively) than common verbena (*Verbena officinalis*) (5.9, 5.8, and 1.50 g/100 g dw, respectively), whereas the opposite trend was recorded for the content of total dietary fibers (70 and 57.1 g/100 g dw in common and lemon verbena, respectively). Moreover, the carbohydrate content did not differ between the species (17.0 and 17.4 g/100 g dw in common and lemon verbena, respectively) while the energetic value was higher in the case of lemon verbena (256 vs. 244 kcal/100 g dw). In contrast to our study, Plaza et al. [60] reported a higher protein content in common verbena leaf extract (12.6 g/100 g dw), which could be due to the different protocol since the authors used the supercritical extraction method while in the present study, the protein content was determined in dried plant tissues. Similarly, Pereira et al. [61,62], who evaluated the nutritional value of dried *A. citrodora* leaves, reported different amounts of macronutrients compared to our study, a finding that indicates the effect of the genotype and growing conditions on the nutritional value of verbena species.

The composition of free sugars, organic acids, and tocopherols is presented in Table 2. Fructose, glucose, and sucrose were the only detected compounds while fructose was the most abundant one in both species. However, a varied content was recorded, with common verbena samples being more abundant than lemon verbena for all the detected individual sugars and consequently total sugars. Sucrose was also detected in *V. officinalis* leaf extracts obtained by supercritical extraction at amounts that varied depending on the extraction temperature (e.g., 42.62 and 37.89 g/100 g dw at 100 and 200 °C, respectively) [60]. Moreover, Pereira et al. [61,62] detected the same main sugars as in our study in *A. citrodora*, although they reported the presence of trehalose, which was not detected in our samples.

In the case of organic acids, succinic acid was the most abundant compound in both species, especially in lemon verbena samples, where the highest content was recorded (3.4 g/100 g dw) (Table 2). Oxalic acid was also present in both species, although its content showed significant differences, with lemon verbena being richer than common verbena (2.46 and 0.36 g/100 g dw in lemon and common verbena, respectively). The citric and malic acid content also differed between the two species, with citric acid being higher in common verbena (1.13 vs. 1.05 g/100 g dw) while malic acid was the most abundant in the case of lemon verbena (1.18 vs. 0.99 g/100 g dw). Finally, quinic acid was only detected in common verbena samples (0.193 g/100 g dw). The same profile of organic acids was reported for *A. citrodora* by Pereira et al. [61,62], although they did not detect succinic acid and they identified shikimic acid instead. Moreover, Guil et al. [63] reported only traces of oxalic acid in *V. officinalis* samples collected in the wild, a finding that is in agreement with our study, where low amounts of oxalic acid were detected.

Regarding tocopherols, α-tocopherol was identified in both species without significant differences being recorded, whereas β-tocopherol was detected only in common verbena samples (Table 2). In contrast to our study, Pereira et al. [61,62] identified three isoforms of vitamin E in *A. citrodora* extracts while α-tocopherol was also the main detected compound in this study.

The fatty acid composition is presented in Table 3. In the case of common verbena, the main fatty acids detected were α-linolenic (36.0%), palmitic (21.1%), linoleic (17.6%), and oleic (13.9%) acids, followed by stearic acid (5.1%), which was detected only in common verbena leaves. In contrast, palmitic acid was the most abundant fatty acid in lemon verbena leaves (32.0%), followed by α-linolenic (24.0%), linoleic (15.7%), oleic (9.8%), and heptadecanoic (8.1%) acids. Moreover, the content of most of the compounds differed between the two species, with lemon verbena recording higher amounts of saturated and monounsaturated fatty acids due to the high contents of palmitic and heptadecanoic acids, whereas common verbena leaves contained higher amounts of polyunsaturated fatty acids (e.g., α-linolenic, linoleic, and oleic acids). Similarly to our study, Guil and Rodríguez-García [64] suggested α-linolenic, linoleic, and palmitic as the main fatty acids in different fractions of *V. officinalis* leaf extracts. On the other hand, Pereira et al. [61] suggested palmitic and linoleic acid as the major fatty acids in *A. citrodora*, whereas they did not detect α-linolenic acid. However, in the study of Pereira et al. [62], α-linolenic acid was the most abundant fatty acid detected in *A. citrodora* extracts, followed by palmitic and linoleic acid.

### 3.2. Chemical Composition

The phenolic compounds content of common and lemon verbena is presented in Table 4. Regarding the contents of total phenolic compounds (TPCs), total flavonoids (TFs), and total phenolic acids (TPAs), varied results were obtained depending on the extraction protocol (e.g., aqueous and hydromethanolic extracts). In particular, the extracts of lemon verbena presented higher amounts of TPCs, TFs, and TPAs than the common verbena extracts. Moreover, aqueous extracts (infusions and decoctions) prepared from both plants were richer in TPCs, TFs, and TPAs than their hydromethanolic ones. For lemon verbena, the TPC values of the present work are in agreement with the findings of Hematian Sourki et al. [47], who studied the chemical composition of alcoholic extracts obtained from Iranian lemon verbena leaves (49.2 mg GAE/g DW), while the TF values were higher in our study (14.5 mg QE/g DW). In contrast, Tammar et al. [65] reported lower amounts of TPCs (from 11.66 to 29.16 mg GAE/g DW) and higher quantities of TFs (from 27.53 to 39.86 mg caffeic acid/g DW) in methanolic extracts obtained from the aerial parts of Tunisian germplasm of lemon verbena. According to Aldeen et al. [66], the TPC and TF content of lemon verbena leaves may vary depending on the plant growth stage, with values ranging between 22.83 and 48.21 and 2.41 and 7.56 mg QE/g for TPCs and TFs, respectively. In the study of Rita et al. [67], the determined amounts of TPCs, TFs, and TPAs in infusions prepared from Portuguese lemon verbena differed from those of our study (230, 117, and 1.91 μg/mL extract), indicating the significant effect that the genotype may have on the phenolic compound composition. In the case of common verbena extracts, Kubica et al. [17], who studied the methanolic extracts of Polish common verbena, recorded a TPC content higher than 100 mg/g DW. The varied reports on lemon verbena and common verbena phenolic compound contents could be attributed to the effects of the growing conditions, harvesting stage and harvesting time, cultivation practices, or extraction protocols [17,66,68,69].

Regarding the phenolic compound profile, nine phenolic compounds were identified and quantified, including seven phenolic acids (gallic (GA), syringic (SRA), *p*-coumaric (*p*CA), ferulic (FA), sinapic (SNA), cinnamic (CA), and protocatechuic (PRA) acids) and two flavonoids (rutin (RUT) and quercetin (Q)) (Table 4). The statistical analysis of the data showed a significant interaction of the tested factor for all the detected phenolic compounds. The content of phenolic compounds showed significant differences between the extracts of common and lemon verbena, with higher amounts of phenolic acids and flavonoids being detected in lemon verbena compared to common verbena extracts. Moreover, in most of the cases, the aqueous extracts of both species were richer in phenolic compounds than their hydromethanolic counterparts. Differences were also recorded in the phenolic compounds profile. In particular, SNA was not detected in all the tested extracts and Q was detected in infusions of common and lemon verbena while PRA was only identified in hydromethanolic extracts of common verbena. Moreover, SRA was detected only in common verbena extracts while *p*CA only in lemon verbena extracts. GA was the most abundant phenolic constituent in all the tested extracts. On the other hand, SRA was detected in higher amounts in common verbena extracts while RUT was the most abundant phenolic compound in lemon verbena extracts. Similarly to our study, Rita et al. [67] also determined 1.91 µg/mL *p*CA in infusions of lemon verbena from Portugal while Aldeen et al. [66] recorded 0.041 mg/g *p*CA and 6.710 mg/g DW FA in lemon verbena leaves, without identifying any amounts of GA and RUT. Finally, Kubica et al. [17] suggested the presence of FA in the range between 8.35 and 29.76 mg/100 g while they recorded 25.75 mg *p*CA/100 g in methanolic extracts of Polish common verbena. These differences could be assigned to different climatic and environmental factors, such as temperature, altitude, soil type, or humidity, where medicinal and aromatic plants are grown [69,70,71]. Moreover, according to Fotakis et al. [72], the preparation method and processing time may result in significant differences in the chemical composition of herbal infusions and decoctions of medicinal plants and could be used as discriminating factors between different species, suggesting that the chlorogenic acid content may be affected by these parameters and present higher contents in infusions than in decoctions. Moreover, Dias et al. [73] reported significant differences in the total and individual phenolic compound contents between hydromethanolic and aqueous (infusions and decoctions) extracts of commercial and wild samples of *Fragaria vesca* roots.

### 3.3. Antioxidant Properties

**Figure 1:**
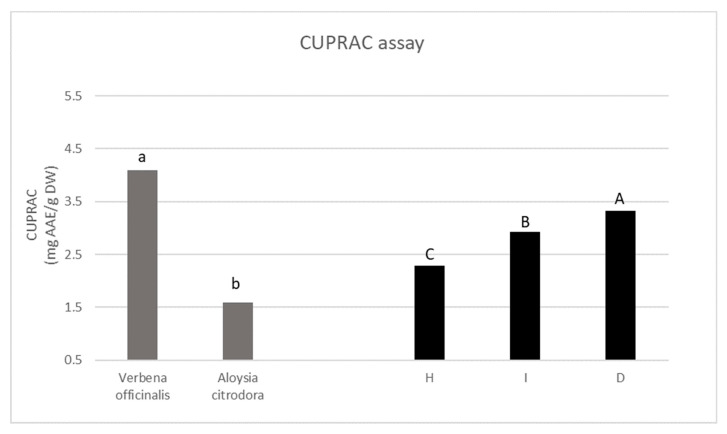
Antioxidant activity of the hydromethanolic extract (H), infusion (I), and decoction (D) of *Verbena officinalis* and *Aloysia citrodora.* Different small letters above each bar indicate significant differences between the extracts of *Verbena officinalis* and *Aloysia citrodora* at *p* < 0.05 while different capital letters above each bar indicate significant differences between the different extracts at *p* < 0.05. Ascorbic acid was used as a positive control (57.85 mg ascorbic acid equivalent (AAE) per g DW).

The antioxidant activity is an important parameter for the quality of food and nutraceutical products. Considering the diversity of mechanisms an antioxidant compound or mixture can exert in vivo, it is inadequate to use a single analytical method to evaluate its in vitro antioxidant capacity [74]. Therefore, it is necessary to apply more than one in vitro chemical-based assay that evaluates the response of the activity of the compound(s) toward reactive oxygen and nitrogen species (ROS/RNS) or other free radicals. The in vitro tests implemented in the present study are appreciated as rapid, simple, low-cost, and reproducible tools for measuring the antioxidant potential of plant extracts [74]. Therefore, they could provide useful information regarding the potential of using the herbal preparation of lemon and common verbena and regarding the preparations that exhibit the highest antioxidant capacity. The statistical analysis of the data showed a significant interaction of the tested factor for all the tested antioxidant activity assays, except for the case of the CUPRAC assay, where only the effect of the main factors (species and extraction) was significant. The results of DPPH, ABTS, and FRAP of the antioxidant activity of the common and lemon verbena extracts are presented in Table 5 while the results of the CUPRAC assay are presented in Figure 1. Generally, lemon verbena samples were characterized by higher antioxidant activity compared to common verbena samples. Moreover, the antioxidant activities of the aqueous extracts of both verbena species were several times higher than those of the hydromethanolic extracts.

Literature data on the antioxidant activity of common and lemon verbena are very scarce and only DPPH and ABTS assays have been determined so far [20,23,32,34]. Although we also included these tests in our study, the recorded DPPH and ABTS values cannot be compared with those obtained by other reports due to the differences in the calculation units. For example, Kubica et al. [17] analyzed methanolic extracts of Polish common verbena and found DPPH values of 0.214 mg/mL (IC_50_) while in decoctions of common verbena, the DPPH values obtained by Babili [75] were 15.76 mg/mL (IC_50_). Moreover, the infusions prepared from Portuguese lemon verbena leaves recorded DPPH values of 0.25 mg/mL (IC_50_) [67] while the DPPH and ABTS values recorded in methanolic extracts of lemon verbena aerial parts from Tunisia were in the range of 12.71 to 14.90 and 4.54 to 8.10 µg/mL (IC_50_), respectively [65]. Plaza et al. [60] suggested that the temperature conditions of supercritical extraction may significantly affect the antioxidant activity of common verbena leaf extracts, with higher values being observed at 200 °C compared to 100 °C (2.156 and 0.959 mmol TE/g dw, respectively). Similarly to our study, the water extracts of Algerian lemon verbena leaves had lower DPPH values (IC_50_ = 27.4 mg/mL) than that of the hydroalcoholic extracts (IC_50_ = 23.52 mg/mL) [76], indicating the effect of the extraction protocol on the recovery of bioactive compounds and consequently on the recorded antioxidant capacity of the extracts. Moreover, Tamer et al. [77], who evaluated the antioxidant activity of lemon juice concentrates fortified with lemon verbena extracts (infusions) with three different methods (namely ABTS, FRAP, and DPPH), reported significant differences between the tested methods. Similar results were reported by Suna et al. [78], who evaluated the antioxidant activity of lemon verbena beverages and suggested significant differences between the DPPH, FRAP, and CUPRAC methods. Dziurka et al. [79] also suggested differences between the total extractable antioxidants and global antioxidant response of lemon verbena extracts determined via the CUPRAC and QUENCHER-CUPRAC assays, respectively. Apart from the genotype and the extraction protocols, the growing conditions may also affect the antioxidant activity of lemon verbena methanolic extracts [80]. To the best of our knowledge, this is the first report regarding the comparison of the antioxidant activity of common and lemon verbena extracts determined with four different methods, including the DPPH, ABTS, FRAP, and CUPRAC assays. Similarly to our study, Dias et al. [73] tested the antioxidant activity of hydrometanolic and aqueous extracts obtained from commercially obtained or wild *Fragaria vesca* roots and suggested that in most antioxidant activity assays (except for thiobarbituric acid reactive substances (TBARSs) and β-carotene inhibition assays in commercial and wild samples, respectively), the aqueous extracts (infusions or decoctions) had higher antioxidant activity than the hydromethanolic extracts. The same trend was also observed in the study of Fotakis et al. [72], who suggested that infusions of *L. citriodora* had higher antioxidant activity (determined with ABTS and FRAP assays) than decoctions. According to the literature, the tested preparations are associated with different conditions (e.g., temperature conditions during the extraction, extraction time) that could affect the release efficiency of particular antioxidant compounds (e.g., phenolic compounds) and consequently regulate the antioxidant capacity of the final extract [72,81].

Regarding the correlation analysis for the tested parameters, the analysis showed 6 and 12 statistically significant correlations (*p* < 0.05) for the common and lemon verbena extracts, respectively (Table 6). In the common verbena extracts, TPA was positively correlated with the DPPH and ABTS values (r^2^ > 0.993). Moreover, FRAP was correlated with TPC (0.984) while DPPH and ABTS radical scavenging activity was highly positively correlated at 0.997. On the other hand, in the lemon verbena extracts, the ABTS radical scavenging activity results were highly correlated with GA, *p*CA, and FA (r^2^ > 0.997) while the FRAP results were correlated with *p*CA (0.996) and ABTS (0.998). According to Chrysargyris et al. [80], the antioxidant activity of lemon verbena extracts is positively correlated with TPC and TF contents, a trend that was also observed in our study. Similar results were reported by Fotakis et al. [72], who recorded a positive correlation between TPC and antioxidant activity in infusions of *L. citriodora* extracts. However, other studies suggest varied results regarding the correlation of TPC and antioxidant activity, depending on the species, the antioxidant activity assay, and the preparation time of the extract [81,82,83]. Considering that the extraction protocol may affect the recovery of antioxidant compounds present in plant matrices [84,85], the recorded differences between the aqueous and hydromethanolic extracts and/or between infusions and decoctions could be associated with the differences in the contents of bioactive molecules in the herbal preparations.

## 4. Conclusions

Common and lemon verbena are popular plants with a wide variety of pharmacological effects. They have been used in herbal medicine as health supplements and/or herbal teas. This study on the nutritional profile, chemical composition, and antioxidant activity of hydromethanolic and aqueous extracts (decoctions and infusions) of common and lemon verbena leaves revealed differences in terms of the nutritional value, chemical composition, and antioxidant activity between the two species. Moreover, a significant positive correlation was recorded between the detected phenolic compounds and the biological activity of the extracts, suggesting the crucial role of phenolic constituents as antioxidant agents in common and lemon verbena extracts. In conclusion, both species showed promising results in terms of the nutritional value, chemical composition, and antioxidant activities, which were positively correlated with the phenolic compound content. Moreover, the extraction protocol may affect the chemical composition and bioactive properties for both species, with the aqueous extracts showing better results than the hydromethanolic ones.

## Figures and Tables

**Table 1:** Parameters of calibrations for the standard phenolic compounds used.

Standards	Regression Equation *	Linearity (µg/mL)	R^2^	LODs (µg/mL)	LOQs (µg/mL)	Recovery (%)
GA	*y* = 33081*x* − 46804	40.4–202	0.984	6.2	20.5	94.53
SRA	*y* = 8297*x* − 19750	40–200	0.993	2.0	5.8	92.47
*p*CA	*y* = 19842*x* − 22834	40–200	0.989	7.3	23.5	94.71
FA	*y* = 40200*x* + 34550	40–200	0.988	5.5	15.1	96.11
SNA	*y* = 39790*x* − 31190	40–200	0.993	4.8	13.6	102.43
CNA	*y* = 80832*x* − 64493	40.8–204	0.996	3.8	10.6	91.59
PRA	*y* = 74574*x* + 57542	40.8–204	0.992	8.5	25.7	104.37
RUT	*y* = 39689*x* + 58775	40–200	0.987	9.2	27.5	95.65
Q	*y* = 66816*x* + 43491	40.8–204	0.991	4.1	12.7	96.83

* *y* is the peak area; *x* refers to the concentration of compounds (µg/mL). GA: gallic acid; SRA: syringic acid; *p*CA: *p*-coumaric acid; FA: ferulic acid; SNA: sinapic acid; CAN: cinnamic acid; PRA: protocatechuic acid; RUT: rutin; Q: quercetin.

**Table 2:** Proximate composition, energy value, and organic acid and tocopherol composition of *Verbena officinalis* and *Aloysia citrodora*.

Constituents (per 100 g)	*Verbena officinalis*	*Aloysia citrodora*
Protein (g)	5.9 ± 0.2 b	13.7 ± 0.5 a
Ash (g)	5.8 ± 0.2 b	9.9 ± 0.2 a
Fat (g)	1.50 ± 0.05 b	1.90 ± 0.07 a
Total dietary fiber (g)	70 ± 1 a	57.1 ± 0.3 b
Available carbohydrates (g)	17 ± 1 a	17.4 ± 0.8 a
Energy (kcal)	244 ± 3 b	256 ± 1 a
Fructose (g)	1.54 ± 0.05 a	1.33 ± 0.02 b
Glucose (g)	0.530 ± 0.006 a	0.390 ± 0.007 b
Sucrose (g)	0.220 ± 0.002 a	0.190 ± 0.002 b
Total free sugars (g)	2.29 ± 0.06 a	1.91 ± 0.02 b
Oxalic acid (g)	0.36 ± 0.02 b	2.46 ± 0.04 a
Quinic acid (g)	0.193 ± 0.006	nd
Malic acid (g)	0.99 ± 0.05 b	1.18 ± 0.08 a
Citric acid (g)	1.13 ± 0.04 a	1.05 ± 0.04 b
Succinic acid (g)	2.24 ± 0.09 b	3.4 ± 0.2 a
Total organic acids (g)	4.92 ± 0.01 b	8.12 ± 0.01 a
α-Tocopherol (mg)	2.58 ± 0.03 a	2.56 ± 0.08 a
β-Tocopherol (mg)	3.23 ± 0.07	nd
Total tocopherols (mg)	5.81 ± 0.04 a	2.56 ± 0.08 b

The results are presented as the mean ± standard deviation on a dry weight (dw) basis. nd: not detected. Different Latin letters in the same row indicate significant differences between the means according to a Student’s *t*-test at *p* < 0.05.

**Table 3:** Fatty acid composition of *Verbena officinalis* and *Aloysia citrodora*.

Fatty Acids (%)	*Verbena officinalis*	*Aloysia citrodora*
Myristic acid (C14:0)	0.85 ± 0.03 b	1.88 ± 0.02 a
Palmitic acid (C16:0)	21.1 ± 0.6 b	32 ± 1 a
Palmitoleic acid (C16:1)	1.06 ± 0.06 a	0.99 ± 0.01 a
Heptadecanoic acid (C17:0)	0.70 ± 0.01 b	1.52 ± 0.04 a
Heptadecenoic acid (C17:1)	0.82 ± 0.04 b	8.1 ± 0.2 a
Stearic acid (C18:0)	5.1 ± 0.2	nd
Oleic acid (C18:1 n9)	13.9 ± 0.5 a	9.8 ± 0.3 b
Linoleic acid (C18:2n6)	17.63 ± 0.01 a	15.7 ± 0.7 b
α-Linolenic acid (C18:3n3)	36 ± 2 a	24.0 ± 0.6 b
Behenic acid (C22:0)	1.69 ± 0.05 b	2.8 ± 0.2 a
Lignoceric acid (C24:0)	1.03 ± 0.03 b	2.74 ± 0.05 a
Fatty acid classes		
Saturated fatty acids (SFAs)	30.5 ± 0.7 b	41 ± 1 a
Monounsaturated fatty acids (MUFAs)	15.8 ± 0.4 b	18.9 ± 0.1 a
Polyunsaturated fatty acids (PUFAs)	54 ± 2 a	39.8 ± 0.1 b

The results are presented as the mean ± standard deviation. nd: not detected. Different Latin letters in the same row indicate significant differences between the means according to a Student’s *t*-test at *p* < 0.05.

**Table 4:** The results of the individual phenolic compound (mg/g DW) and total flavonoid (TF), phenolic acid (TPA) and phenolic content (TPC) of the hydromethanolic extract, infusion, and decoction of *Verbena officinalis* and *Aloysia citrodora*.

	*Verbena officinalis*			*Aloysia citrodora*		
	Hydromethanolic Extract	Infusion	Decoction	Hydromethanolic Extract	Infusion	Decoction
GA	0.06 ± 0.01 a	0.34 ± 0.15 c	0.77 ± 0.32 d	0.14 ± 0.06 b	2.74 ± 0.95 e	2.82 ± 0.63 ef
SRA	1.03 ± 0.41 a	2.61 ± 0.16 b	2.69 ± 0.17 b	nd	nd	nd
*p*CA	nd	nd	nd	0.05 ± 0.02 a	0.15 ± 0.01 b	0.17 ± 0.01 bc
FA	0.24 ± 0.09 a	1.80 ± 0.14 b	2.03 ± 0.34 b	0.59 ± 0.10 a	5.88 ± 0.65 c	6.27 ± 0.32 c
SNA	nd	nd	nd	nd	nd	nd
CNA	0.02 ± 0.00 a	0.09 ± 0.01 ab	5.12 ± 1.43 c	0.03 ± 0.00 a	0.12 ± 0.01 b	0.12 ± 0.09 b
PRA	nd	1.35 ± 0.04 a	1.36 ± 0.04 a	0.78 ± 0.17 c	2.77 ± 0.20 b	2.74 ± 0.04 b
RUT	0.86 ± 0.47 b	1.90 ± 0.25 a	1.75 ± 0.16 a	4.96 ± 1.17 d	7.79 ± 0.45 e	3.32 ± 0.18 c
Q	0.54 ± 0.01 a	nd	4.49 ± 0.08 b	0.54 ± 0.02 a	nd	4.51 ± 0.08 b
TF (mg QE/g DW)	1.15 ± 0.33 a	1.65 ± 0.29 a	3.15 ± 0.16 b	2.18 ± 0.34 c	3.23 ± 0.57 b	5.04 ± 0.30 d
TPA (mg CE/g DW)	1.56 ± 0.09 a	11.60 ± 1.42 c	15.14 ± 0.51 d	5.69 ± 0.56 b	38.13 ± 1.77 f	28.51 ± 11.14 e
TPC (mg GE/g DW)	25.45 ± 3.81 a	77.53 ± 3.01 c	104.64 ± 6.77 d	49.30 ± 1.77 b	137.46 ± 5.17 e	193.49 ± 7.55 f

The results in the same row followed by the same small letters do not significantly differ by the Tukey test (*p* < 0.05); nd: not detectable. GA: gallic acid; SRA: syringic acid; *p*CA: *p*-coumaric acid, FA: ferulic acid; SNA: sinapic acid; CNA: cinnamic acid; PRA: protocatechuic acid; RUT: rutin; Q: quercetin.

**Table 5:** Antioxidant activity of the hydromethanolic extract, infusion, and decoction of *Verbena officinalis* and *Aloysia citrodora*.

	*Verbena officinalis*			*Aloysia citrodora*			
	Hydromethanolic Extract	Infusion	Decoction	Hydromethanolic Extract	Infusion	Decoction	Ascorbic Acid
DPPH (mg TE/g DW)	25.90 ± 7.39 a	111.35 ± 9.12 c	134.25 ± 2.65 d	77.11 ± 6.95 b	219.10 ± 2.57 e	280.22 ± 2.07 f	68.45 ± 1.32
ABTS (mg TE/g DW)	19.04 ± 5.91 a	132.57 ± 4.58 c	162.59 ± 4.62 d	48.88 ± 1.19 b	342.99 ± 8.52 e	374.77 ± 5.57 f	32.68 ± 1.12
FRAP (mmol Fe^2+^/g DW)	0.37 ± 0.07 a	3.89 ± 0.12 c	5.91 ± 0.50 d	0.91 ± 0.01 b	7.31 ± 0.41 e	8.45 ± 1.01 f	1.74 ± 0.34

Means in the same row followed by the same letters do not significantly differ according to Tukey’s HSD test (*p* < 0.05); Ascorbic acid was used as a positive control of the DPPH, ABTS, FRAP, and CUPRAC tests.

**Table 6:** Correlation coefficient analysis of the phenolic composition and antioxidant activity in *Verbena officinalis* and *Aloysia citrodora* samples.

	*Verbena officinalis*				*Aloysia citrodora*			
	DPPH	ABTS	FRAP	CUPRAC	DPPH	ABTS	FRAP	CUPRAC
GA	0.903	0.702	0.962	0.910	0.903	0.998 *	0.793	0.750
SRA	0.917	0.887	0.947	0.936	-	-	-	-
*p*CA	-	-	-	-	0.890	0.997 *	0.996 *	0.882
FA	0.964	0.896	0.968	0.940	0.772	0.999 *	0.883	0.860
CNA	0.672	0.670	0.785	0.805	0.856	0.796	0.890	0.741
PRA	0.880	0.781	0.935	0.823	0.852	0.894	0.788	0.637
RUT	0.844	0.744	0.876	0.860	−0.144	0.064	0.012	−0.189
Q	0.577	0.575	0.704	0.727	0.652	0.481	0.524	0.686
TF	0.823	0.822	0.906	0.909	0.929	0.832	0.860	0.845
TPA	0.996 *	0.998*	0.992	0.882	0.830	0.828	0.907	0.804
TPC	0.909	0.880	0.997 *	0.896	0.795	0.853	0.776	0.798

* indicates significance at *p* < 0.05 level.

## Data Availability

Data is contained within the article.
